# Supplementary material for: Inverted battery design as ion generator for interfacing with biosystems
Source: Nat Commun. 2017 Jul 24;8:15609. doi: 10.1038/ncomms15609 (PMC5527283; doi:10.1038/ncomms15609)
Supplement: Supplementary Information — Supplementary Figures and Supplementary Methods [file ncomms15609-s1.pdf]

Supplementary Figures

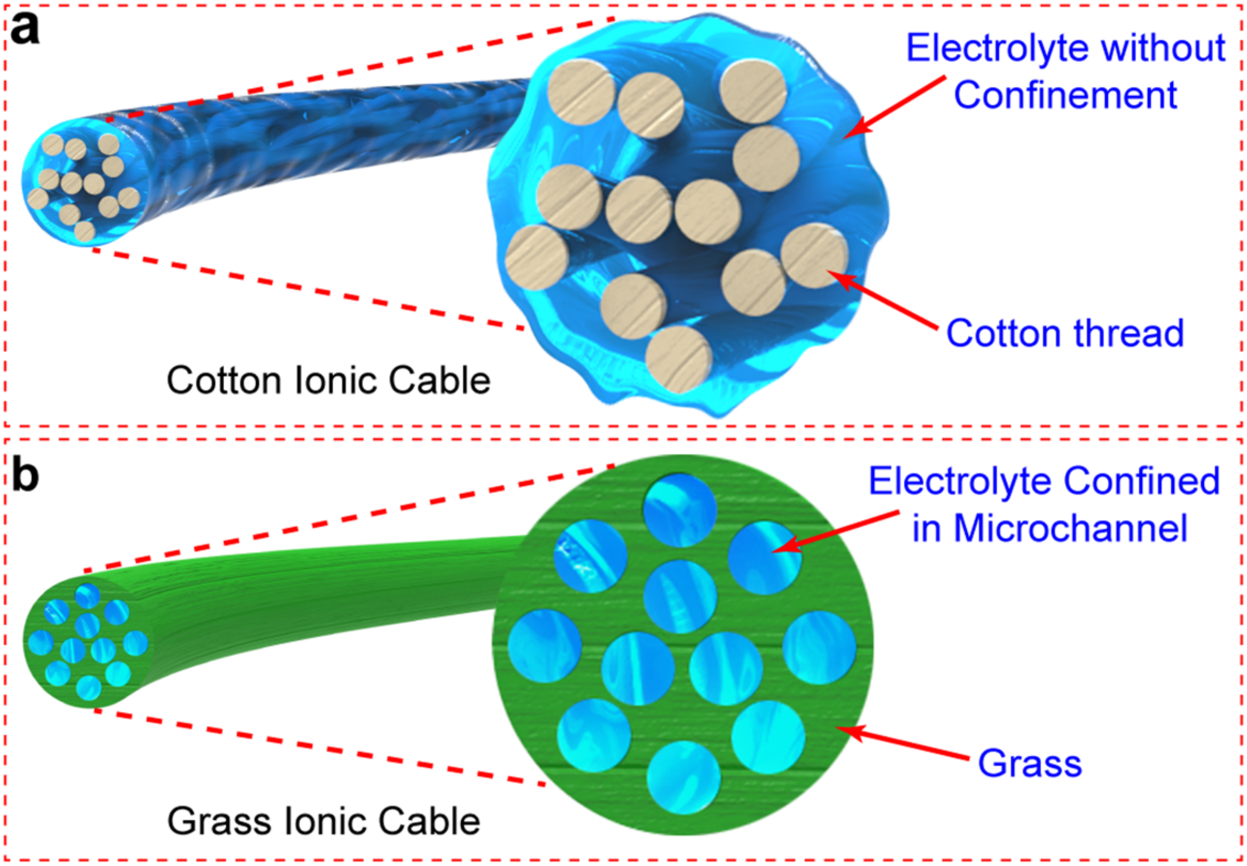

Supplementary Figure 1. The illustration of ionic cables. (**a**) The cotton ionic cable is made of an electrolyte-soaked cotton string, where the electrolyte surrounds the cotton fiber. (**b**) In the grass ionic cable, the electrolyte is stored within the micro-aligned grass channels, which prevents electrolyte loss.

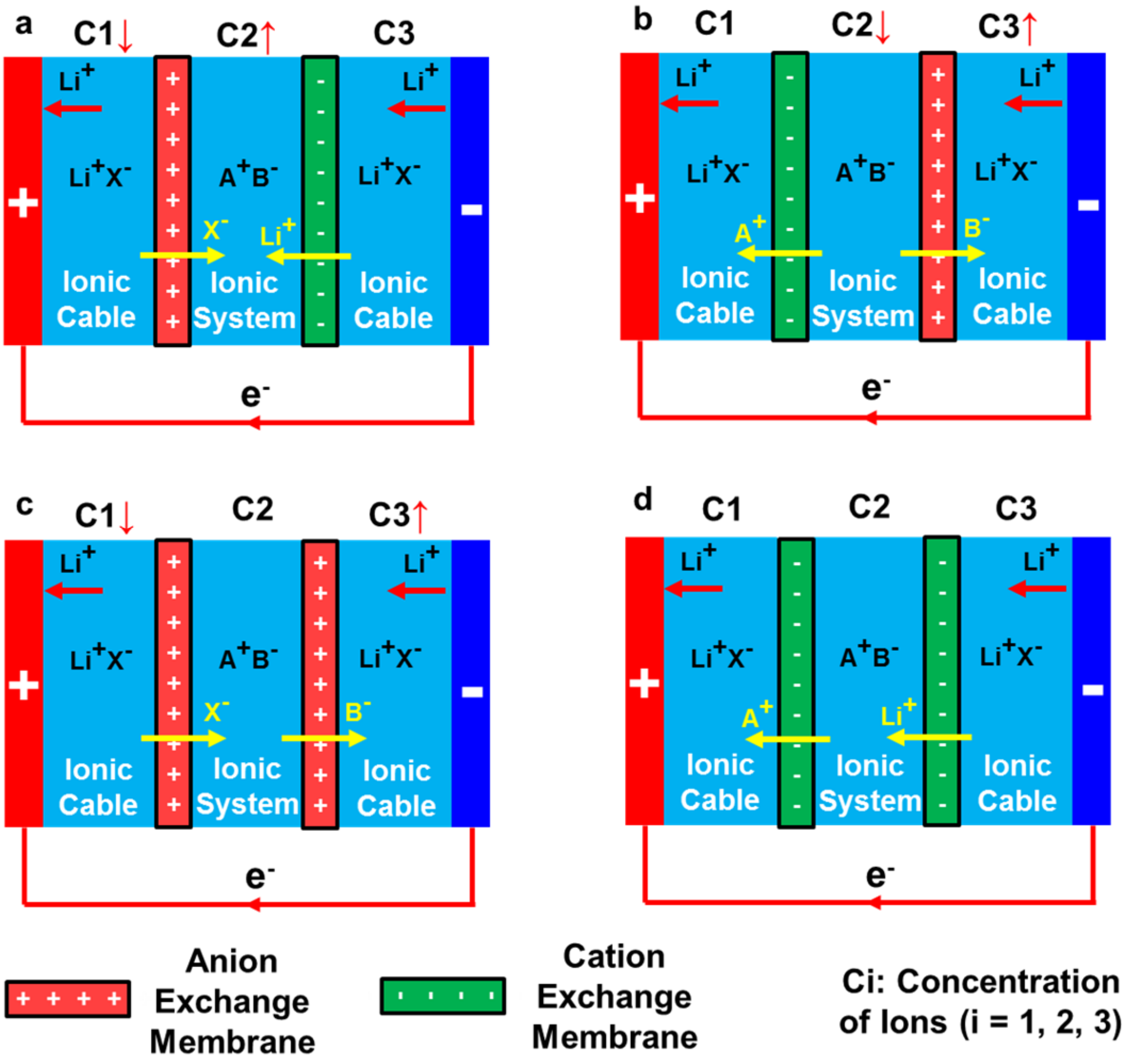

Supplementary Figure 2. The change of ion concentration within different ion exchange membrane sections. (**a**) When an AEM and CEM are used as the cathode and anode, respectively, the ion concentration on the cathode ionic cable side will decrease, while the ion concentration in the ionic system will increase. (**b**) When the AEM and CEM are reversed, the ion concentration on the anode side of the ionic cable will increase, while the ion concentration in the ionic system will decrease. (**c**) When an AEM is used for both electrodes, the ion concentration on the cathode side of the ionic cable will decrease, while the ion concentration on the anode side of the ionic cable will increase. (**d**) When both electrodes are CEMs, the ion concentration in each part stays constant.

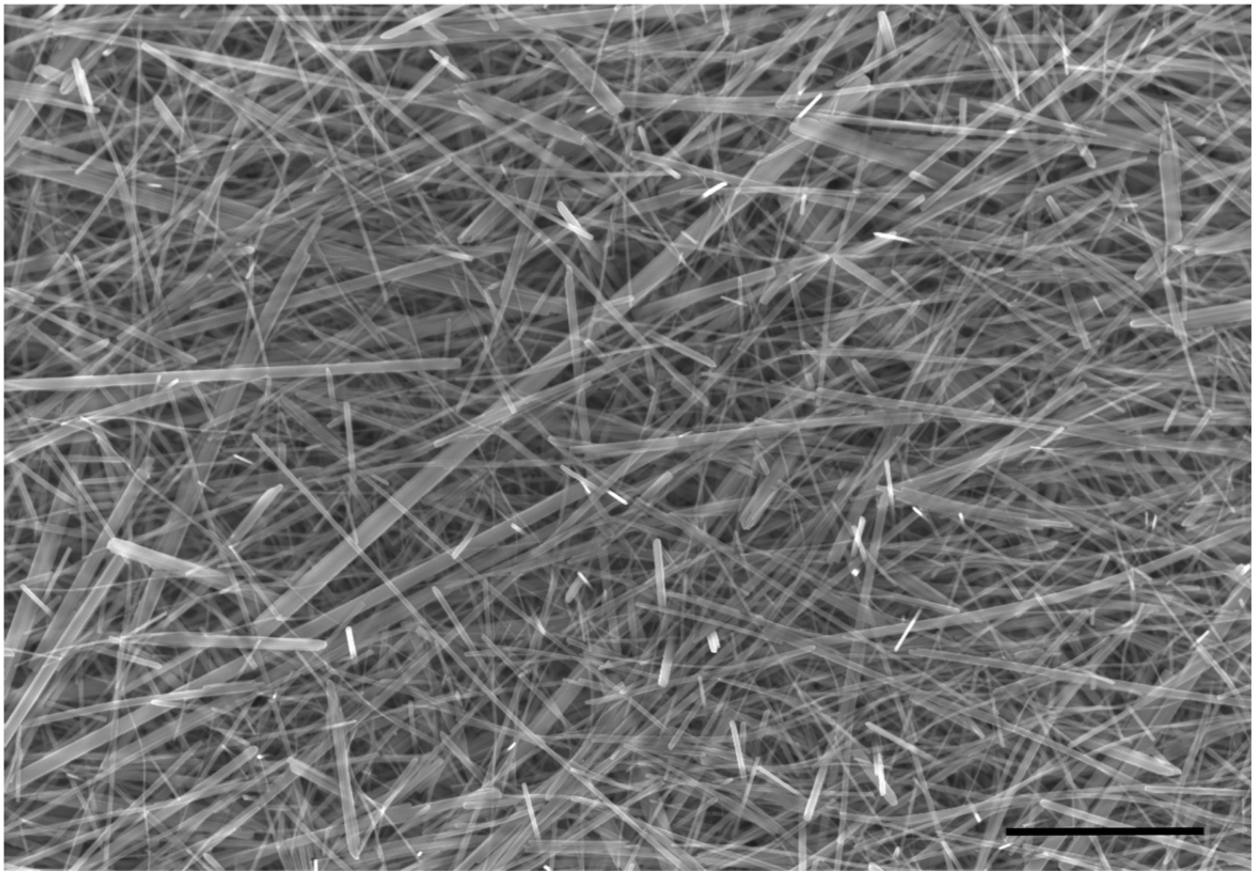

Supplementary Figure 3. The SEM image of V_2O_5 nanowires synthesized by hydrothermal method. Scale bar, 2 μm.

Supplementary Methods

Any traditional battery materials (anode, cathode, organic electrolytes) have potential in our inverted battery design due to similarities in the electrochemical process.

Lithium (Li) metal was selected as the anode in this work to achieve a high cell voltage since Li has the highest reduction potential (-3.04 V). As we mentioned in the manuscript, the electron battery has potential as the ion source to interact with bio-systems. For these specific applications, Li, Na, K, Ca, *et al.* can be selected as the anode materials to generate Li^+, Na^+, K^+, Ca^{2+}, respectively. For example, lithium has been used as one of the most effective treatment methods for bipolar disorder. If an implantable electron battery with a lithium anode acted as the Li^+ source, lithium can be controllably released as needed, which could be a more effective and safer way to treat bipolar disorder.

The cathodes were chosen according to the specific anode material used. In this work, V_2O_5 nanowires acted as the cathode to enable fast Li^+ diffusion and high discharge rates. These properties are achieved due to the nanostructure of the V_2O_5. Moreover, V_2O_5 starts from the non-lithiated state, therefore, no initial charge process was needed to use the electron battery. Note that other cathode materials used in traditional lithium ion batteries can also be employed here.

The electrolyte was selected based on the electrode materials. Here, the commonly used $LiPF_6$ in EC/DEC (1:1 by volume) electrolyte was used for the Li metal anode and its corresponding cathode material. Note that other Li^+-based electrolytes can also be used with Li metal anodes. If Na, K, *et al.* were used as the anode material, the corresponding electrolytes would contain Na^+ or K^+, respectively.

The main function of the ion exchange membranes is to prevent the organic electrolyte within

the electrodes from mixing with the aqueous electrolytes within the ionic cables. Therefore, a membrane with low solvent permeability is preferred. A high ionic conductivity is also preferred to lower the working resistance. The ion exchange membranes consisted of two types: anion exchange membranes (AEMs) and cation exchange membrane (CEMs). The combination of these membranes also results in different behavior. The diagram in Supplementary Figure 2 shows that different membrane combinations cause drastic ion concentration changes. In some configurations, these sections act as an ionic pump to selectively change the local ion concentrations. In this work, two CEMs were used for both electrodes (Supplementary Figure 2d), therefore, there is no local ion concentration change.
